# PAX8 expression in cancerous and non-neoplastic tissue: a tissue microarray study on more than 17,000 tumors from 149 different tumor entities

**DOI:** 10.1007/s00428-024-03872-y

**Published:** 2024-08-06

**Authors:** Natalia Gorbokon, Sarah Baltruschat, Maximilian Lennartz, Andreas M. Luebke, Doris Höflmayer, Martina Kluth, Claudia Hube-Magg, Andrea Hinsch, Christoph Fraune, Patrick Lebok, Christian Bernreuther, Guido Sauter, Andreas H. Marx, Ronald Simon, Till Krech, Till S. Clauditz, Frank Jacobsen, Eike Burandt, Stefan Steurer, Sarah Minner

**Affiliations:** 1 Institute of Pathology, University Medical Center Hamburg-Eppendorf, Hamburg, Germany; 2 Institute of Pathology, Clinical Center Osnabrueck, Osnabrueck, Germany; 3 Department of Pathology, Academic Hospital Fuerth, Fuerth, Germany

**Keywords:** PAX8, Immunohistochemistry, Diagnostic marker, Tissue microarray, Cancer

## Abstract

PAX8 plays a role in development of the thyroid, kidney, and the Wolffian and Mullerian tract. In surgical pathology, PAX8 immunohistochemistry is used to determine tumors of renal and ovarian origin, but data on its expression in other tumors are conflicting. To evaluate PAX8 expression in normal and tumor tissues, a tissue microarray containing 17,386 samples from 149 different tumor types and 608 samples of 76 different normal tissue types was analyzed by immunohistochemistry. PAX8 results were compared with previously collected data on cadherin 16 (CDH16). PAX8 positivity was found in 40 different tumor types. The highest rate of PAX8 positivity was found in thyroidal neoplasms of follicular origin (98.6–100%), gynecological carcinomas (up to 100%), renal tumors (82.6–97.8%), and urothelial neoplasms (2.3–23.7%). Important tumors with near complete absence of PAX8 staining (< 1%) included all subtypes of breast cancers, hepatocellular carcinomas, gastric, prostatic, pancreatic, and pulmonary adenocarcinomas, neuroendocrine neoplasms, small cell carcinomas of various sites, and lymphomas. High PAX8 expression was associated with low tumor grade in 365 non-invasive papillary urothelial carcinomas (*p* < 0.0001) but unrelated to patient outcome and/or tumor phenotype in clear cell renal cell carcinoma, high-grade serous ovarian cancer, and endometrioid endometrial carcinoma. For determining a renal tumor origin, sensitivity was 88.1% and specificity 87.2% for PAX8, while sensitivity was 85.3% and specificity 95.7% for CDH16. The combination of PAX8 and CDH16 increased specificity to 96.8%. In conclusion, PAX8 immunohistochemistry is a suitable diagnostic tool. The combination of PAX8 and CDH16 positivity has high specificity for renal cell carcinoma.

**Supplementary Information:**

The online version contains supplementary material available at 10.1007/s00428-024-03872-y.

## Introduction

PAX8 is a member of the paired-box gene family and is expressed in embryogenesis and organ development of the thyroid, Mullerian, Wolffian, and renal/upper urinary tract and is also required for tissue homeostasis in the respective adult tissues [25, 32]. PAX8 is a transcriptional regulator of thyroid-specific genes such as thyroglobulin, thyroid peroxidase, and the sodium-iodide symporter by binding to promoter regions through its 128-amino acid paired domain and is essential for thyroid follicular cell metabolism [9, 53]. In Wolffian and Mullerian duct derived tissues, PAX8 is important for mesenchymal-to-epithelial transition (EMT), regulates branching morphogenesis and nephron differentiation [28, 49], and may also modulate WT1 transcription [10].

In diagnostic pathology, PAX8 immunohistochemistry (IHC)—in combination with other markers—is often used to determine the origin of tumors that are difficult to classify by morphology alone. Detectable PAX8 expression is considered a strong argument for a tumor origin from the kidney, thyroid, or inner female genital tract [30, 43]. Numerous reports have described PAX8 expression in cancer. For many tumor types, however, the reported frequencies of PAX8 positivity vary considerably, which makes it practically impossible to derive the prevalence of PAX8 expression in a particular tumor type from the literature. For example, the reported rate of PAX8 positivity ranges from 0 to 100% in cervical squamous cell carcinoma [20, 30], anaplastic thyroid cancer [35, 48], and non-invasive and invasive urothelial cancer [33, 46], from 0 to 96% in Merkel cell carcinoma [22, 38], from 0 to 83% in cervical adenocarcinoma [43, 46], from 0 to 75% in medullary thyroid cancer [22, 29], from 31 to 100% in papillary thyroid carcinoma [20, 54], from 42 to 95% in renal oncocytoma [46, 55], and from 38 to 100% in endometrioid adenocarcinoma of the ovary [12, 18]. These conflicting data are probably caused by the use of different antibodies, immunostaining protocols, and criteria to determine PAX8 positivity in these studies.

To better understand the prevalence and diagnostic utility of PAX8 immunostaining in cancer, a comprehensive study analyzing a large number of neoplastic and non-neoplastic tissues under highly standardized conditions is desirable. Therefore, PAX8 expression was analyzed in more than 17,000 tumor tissue samples from 149 different tumor types and subtypes as well as 76 non-neoplastic tissue categories by IHC in a tissue microarray (TMA) format in this study.

## Materials and methods

### Tissue microarrays (TMAs)

Our normal tissue TMA was composed of 8 samples from 8 different donors for each of 76 different normal tissue types (608 samples on one slide). The cancer TMAs contained a total of 17,386 primary tumors from 149 tumor types and subtypes. Detailed histopathological and molecular data were available for cancers of the kidney (*n* = 1757), ovary (*n* = 524), endometrium (*n* = 259), and the bladder (*n* = 1663). Clinical follow-up data were accessible from 850 renal cell cancer patients with a median follow-up time of 39 months. Data on the expression of cadherin 16 (CDH16) [21], GATA3 [36], and p63 [41] were available from previous studies using subsets of the TMAs of this study. The composition of normal and cancer TMAs is described in the results section. All samples were from the archives of the Institutes of Pathology, University Hospital of Hamburg, Germany, the Institute of Pathology, Clinical Center Osnabrueck, Germany, and Department of Pathology, Academic Hospital Fuerth, Germany. Tissues were fixed in 4% buffered formalin and then embedded in paraffin. The TMA manufacturing process was described earlier in detail [8, 19]. In brief, one tissue spot (diameter 0.6 mm) was transmitted from a tumor containing donor block to an empty recipient paraffin block. The use of archived remnants of diagnostic tissues for TMA manufacturing, their analysis for research purposes, and patient data were according to local laws (HmbKHG, §12), and analysis had been approved by the local ethics committee (Ethics Commission Hamburg, WF-049/09). All work has been carried out in compliance with the Helsinki Declaration.

### Immunohistochemistry

Freshly prepared TMA sections were immunostained on one day in one experiment. Slides were deparaffinized with xylol, rehydrated through a graded alcohol series, and exposed to heat-induced antigen retrieval for 5 min in an autoclave at 121 °C in pH 7.8 DakoTarget Retrieval Solution™ (Agilent, CA, USA; #S2367). Endogenous peroxidase activity was blocked with Dako Peroxidase Blocking Solution™ (Agilent, CA, USA; #S2023) for 10 min. Primary antibody specific for PAX8 (rabbit recombinant, MSVA-708R, MS Validated Antibodies, GmbH, Hamburg, Germany; #3331-708R) was applied at 37 °C for 60 min at a dilution of 1:150. For the purpose of antibody validation, the normal tissue TMA was also analyzed by the mouse monoclonal PAX8 antibody MRQ-50 (Cell Marque™—Sigma Aldrich®, CA, USA; #363 M) at a dilution of 1:15 and an otherwise identical protocol. Bound antibody was visualized using the EnVision Kit™ (Agilent, CA, USA; #K5007) according to the manufacturer’s directions. A subset of 1009 tumors (as detailed in supplementary Fig. 4) were also analyzed with both antibodies to document the impact of antibody selection on staining results. The sections were counterstained with hemalaun. For tumor tissues, the percentage of positive neoplastic cells was estimated, and the staining intensity was semiquantitatively recorded (0, 1 + , 2 + , and 3 +). For statistical analyses, the staining results were categorized into four groups. Tumors without any staining were considered negative. Tumors with 1 + staining intensity in ≤ 70% of tumor cells or 2 + intensity in ≤ 30% of tumor cells were considered weakly positive. Tumors with 1 + staining intensity in > 70% of tumor cells, 2 + intensity in 31–70%, or 3 + intensity in ≤ 30% of tumor cells were regarded as moderately positive. Tumors with 2 + intensity in > 70% or 3 + intensity in > 30% of tumor cells were considered strongly positive.

### Statistics

Statistical calculations were performed with JMP® 16 software (SAS Institute Inc., NC, USA). Contingency tables and the chi^2^-test were performed to search for associations between PAX8 and tumor phenotype. Survival curves were calculated according to Kaplan–Meier. The Log-Rank test was applied to detect significant differences between groups. Sensitivity and specificity were calculated using the formulas TP/(TP + FN) and TN/(TN + FP), respectively, where TP is the number of true positive, TN is the number of true negative, FP is the number of false positive, and FN is the number of false negative.

## Results

### Technical issues

A total of 15,223 (87.6%) of 17,386 tumor samples were interpretable in our TMA analysis. Non-interpretable samples demonstrated absence of unequivocal tumor cells or a complete lack of individual tissue spots. A sufficient number of samples of each normal tissue type was always evaluable (≥ 4).

### PAX8 in normal tissues

**Fig. 1:**
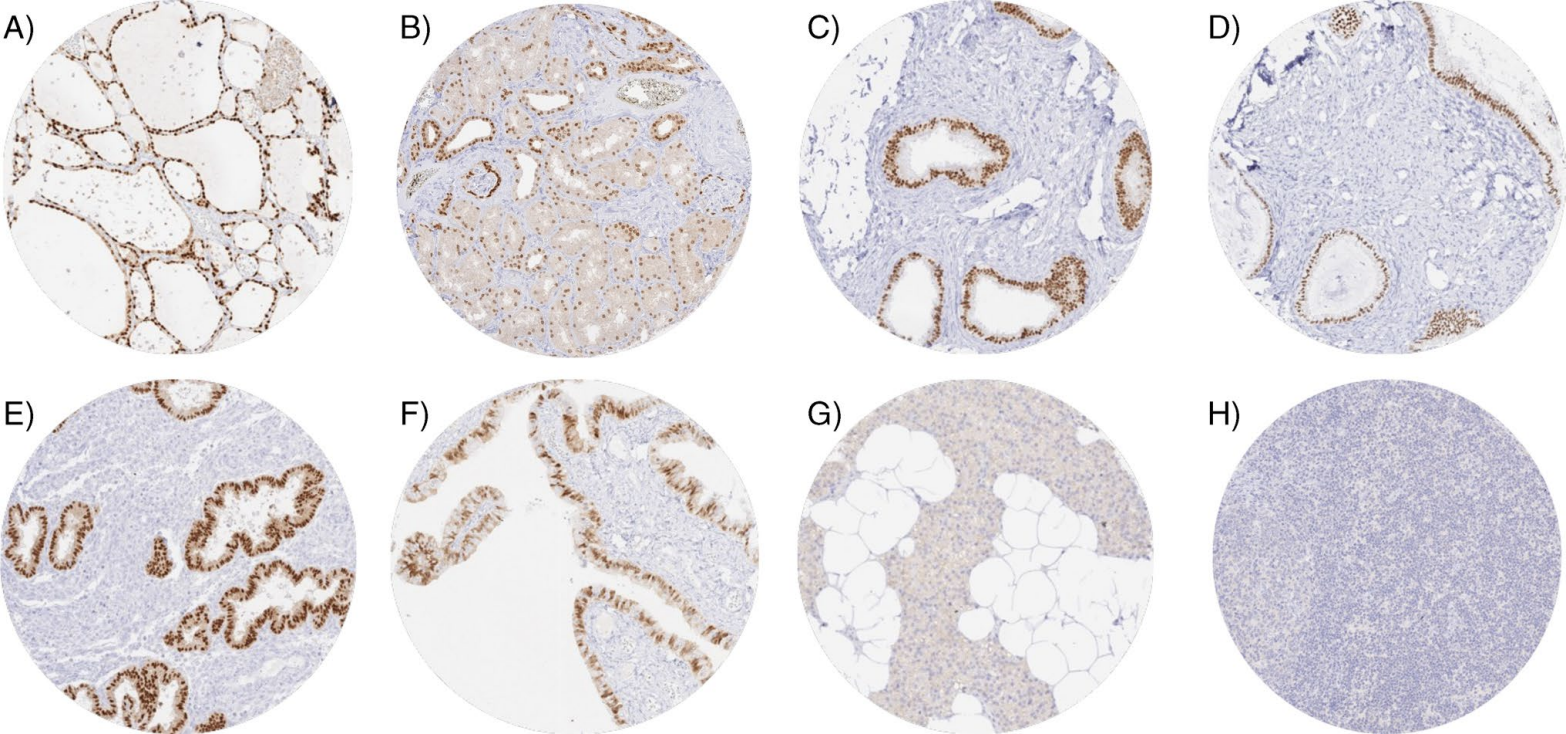
Pattern of PAX8 immunostaining in normal tissues. The panels show a strong PAX8 staining in distinct cell types of the thyroid (**A**), kidney (**B**), caput epididymis (**C**), endocervical mucosa (**D**), endometrium (**E**), and the fallopian tube (**F**). PAX8 staining is absent in a parathyroid gland (**G**) and a lymph node (**H**)

A strong nuclear PAX8 staining, which was often accompanied by a weak cytoplasmic staining, was observed in follicular cells of the thyroid, epithelial cells of the endometrium, endocervix, and the epididymis, ciliated epithelial cells of the fallopian tube, and a subset of epithelial cells of the seminal vesicle. In the kidney, variable, weak to strong nuclear PAX8 staining of cells was seen in proximal and distal tubuli, collecting ducts and epithelial cells of the parietal membrane of the Bowman’s capsule. In some samples, a weak to moderate nuclear PAX8 staining was also seen in basal and suprabasal cell layers of the urothelium, especially in the renal pelvis. Representative images of PAX8 staining are given in Fig. 1. All these nuclear stainings were observed by both MRQ-50 and MSVA-708R. Additional nuclear staining of a subset of lymphocytes, thymic epithelial cells, pancreatic islet cells, epithelial cells of the parathyroid, and neuroendocrine cells of the gastrointestinal tract as well as a granular cytoplasmic staining in acinar cells of the pancreas and in other cells was only seen by MRQ-50 and thus considered antibody specific cross-reactivities of MRQ-50. An additional, purely cytoplasmic staining in a small subset of inflammatory cells of the intestine, gallbladder epithelium, in some samples of gastric glands, and a subset of epithelial cells of the adenohypophysis was only observed by MSVA-708R and thus considered an antibody specific cross-reactivity of MSVA-708R. Comparative images showing staining by both antibodies are given in supplementary Fig. 1. PAX8 staining was absent in intima and media of the aorta, heart (left ventricle), skeletal muscle, skeletal muscle/tongue, myometrium, muscular wall of the gastro-intestinal-tract (appendix, esophagus, stomach, ileum, and colon descendens), muscular wall of the renal pelvis and bladder, ovarian stroma, keratinocytes of the epidermis, sebaceous glands, squamous epithelium of the ectocervix, placental cells (cytotrophoblast, syncytiotrophoblast, amnion, and chorion), decidua, gastric epithelial cells, enterocytes of the small and large intestine including appendix, hepatocytes, Kupffer cells, acinic cells and ductal cells of the exocrine pancreas, mucinous and/or serous epithelium as well as ductal cells of the salivary glands (parotis, glandular submandibularis, and glandular sublingualis), Sertoli cells, Leydig cells, and germinal cells of the testis, bronchus epithelium, pneumocytes, epithelium of the paranasal sinus, glandular and ductal epithelium of the breast, cortical and medullary cells of the adrenal gland, neuronal and glial cells of the cerebrum and cerebellum, and in cells of the neurohypophysis.

### PAX8 in cancer

**Fig. 2:**
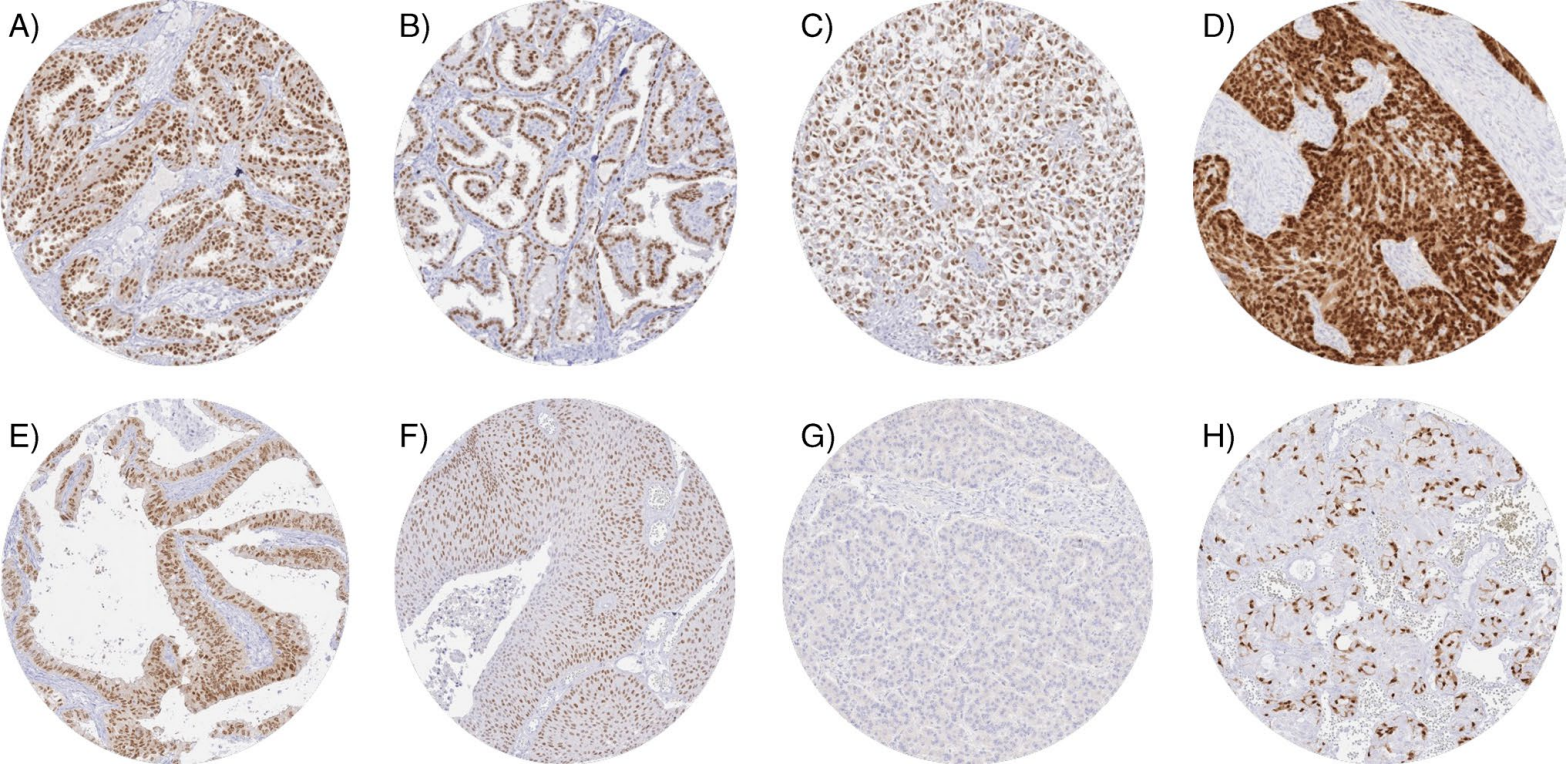
PAX8 immunostaining in cancer. PAX8 staining is predominantly nuclear but accompanied by a weaker cytoplasmic positivity in cases with strong positivity. The panels show PAX8 positivity in a papillary renal cell carcinoma (**A**), a papillary (**B**), and an anaplastic (**C**) thyroid cancer, a serous high-grade ovarian carcinoma (**D**), an adenocarcinoma of the cervix (**E**), and a non-invasive papillary urothelial carcinoma (**F**). PAX8 staining is absent in a neuroendocrine tumor of the pancreas (**G**) and in a medullary carcinoma of the thyroid containing entrapped PAX8-positive follicular epithelial cells (**H**)

**Fig. 3:**
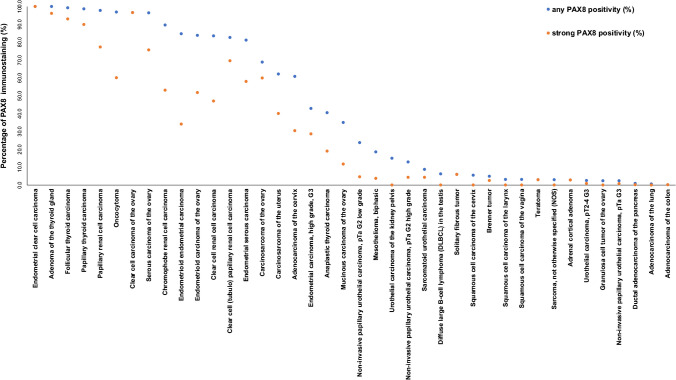
Ranking order of PAX8 immunostaining in tumors. Both the percentage of positive cases (blue dots) and the percentage of strongly positive cases (orange dots) are shown

**Fig. 4:**
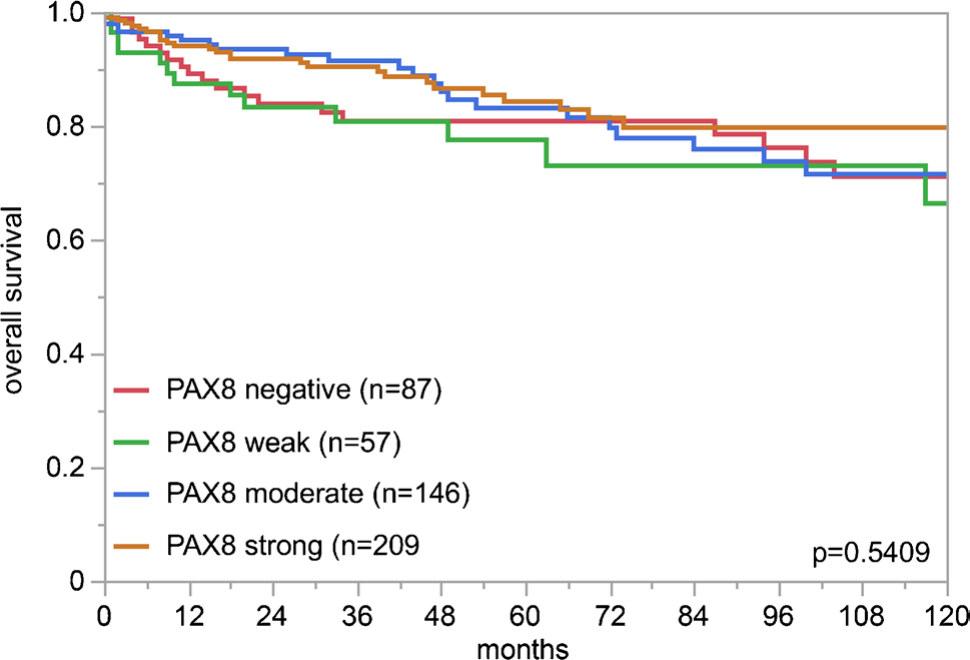
PAX8 immunostaining and patient prognosis in clear cell renal cell carcinoma

PAX8 immunostaining was detectable in 3,400 (22.3%) of the 15,223 analyzable tumors, including 330 (2.2%) with weak, 782 (5.1%) with moderate, and 2,288 (15.0%) with strong immunostaining. Overall, 40 (26.8%) of 149 tumor categories showed detectable PAX8 expression with 32 (21.5%) tumor categories including at least one case with strong positivity (Table 1). Representative images of PAX8-positive tumors are shown in Fig. 2. The highest rate of PAX8 positivity was found in thyroidal neoplasms of follicular origin (98.6–100%), gynecological adenocarcinomas (up to 100%), renal tumors (82.6–97.8%), and urothelial neoplasms (2.3–23.7%). A graphical representation of a ranking order of PAX8 positive and strongly positive cancers is given in Fig. 3. Clinically relevant tumors with near complete absence of PAX8 staining (< 1%) included for example all subtypes of breast cancers, gastric adenocarcinoma, prostatic adenocarcinoma, hepatocellular carcinomas, cholangiocarcinoma, gallbladder adenocarcinoma, pulmonary adenocarcinoma, neuroendocrine neoplasms, and small cell carcinomas of various sites. High PAX8 expression was associated with low tumor grade in a cohort of 365 non-invasive papillary urothelial carcinomas (*p* < 0.0001) but unrelated to patient outcome and/or unfavorable tumor phenotype in clear cell renal cell carcinoma, high-grade serous ovarian cancer, and endometrioid endometrium carcinoma (Table 2 and Fig. 4).

**Table 1:** PAX8 immunostaining in human tumors

	Tumor entity	On TMA (n)	PAX8 immunostaining				
			n*	Neg. (%)	Weak (%)	Mod. (%)	Str. (%)
Tumors of the skin	Basal cell carcinoma	89	77	100.0	0.0	0.0	0.0
	Benign nevus	29	23	100.0	0.0	0.0	0.0
	Squamous cell carcinoma of the skin	145	129	100.0	0.0	0.0	0.0
	Malignant melanoma	65	58	100.0	0.0	0.0	0.0
	Malignant melanoma lymph node metastasis	86	86	100.0	0.0	0.0	0.0
	Merkel cell carcinoma	2	2	100.0	0.0	0.0	0.0
Tumors of the head and neck	Squamous cell carcinoma of the larynx	109	98	96.9	2.0	1.0	0.0
	Squamous cell carcinoma of the pharynx	60	60	100.0	0.0	0.0	0.0
	Oral squamous cell carcinoma (floor of the mouth)	130	125	100.0	0.0	0.0	0.0
	Pleomorphic adenoma of the parotid gland	50	37	100.0	0.0	0.0	0.0
	Warthin tumor of the parotid gland	104	82	100.0	0.0	0.0	0.0
	Adenocarcinoma, NOS (papillary cystadenocarcinoma)	14	11	100.0	0.0	0.0	0.0
	Salivary duct carcinoma	15	9	100.0	0.0	0.0	0.0
	Acinic cell carcinoma of the salivary gland	181	108	100.0	0.0	0.0	0.0
	Adenocarcinoma NOS of the salivary gland	109	56	100.0	0.0	0.0	0.0
	Adenoid cystic carcinoma of the salivary gland	180	69	100.0	0.0	0.0	0.0
	Basal cell adenocarcinoma of the salivary gland	25	19	100.0	0.0	0.0	0.0
	Basal cell adenoma of the salivary gland	101	54	100.0	0.0	0.0	0.0
	Epithelial-myoepithelial carcinoma of the salivary gland	53	42	100.0	0.0	0.0	0.0
	Mucoepidermoid carcinoma of the salivary gland	343	267	100.0	0.0	0.0	0.0
	Myoepithelial carcinoma of the salivary gland	21	15	100.0	0.0	0.0	0.0
	Myoepithelioma of the salivary gland	11	9	100.0	0.0	0.0	0.0
	Oncocytic carcinoma of the salivary gland	12	5	100.0	0.0	0.0	0.0
	Polymorphous adenoca. low grade, of the salivary gland	41	13	100.0	0.0	0.0	0.0
	Pleomorphic adenoma of the salivary gland	53	33	100.0	0.0	0.0	0.0
Tumors of the lung, pleura, and thymus	Adenocarcinoma of the lung	196	180	99.4	0.0	0.6	0.0
	Squamous cell carcinoma of the lung	80	67	100.0	0.0	0.0	0.0
	Mesothelioma, epithelioid	40	33	100.0	0.0	0.0	0.0
	Mesothelioma, biphasic	29	27	81.5	14.8	0.0	3.7
	Thymoma	29	24	100.0	0.0	0.0	0.0
	Lung, neuroendocrine tumor (NET)	29	25	100.0	0.0	0.0	0.0
Tumors of the female genital tract	Squamous cell carcinoma of the vagina	78	66	97.0	3.0	0.0	0.0
	Squamous cell carcinoma of the vulva	157	145	100.0	0.0	0.0	0.0
	Squamous cell carcinoma of the cervix	136	129	94.6	3.1	2.3	0.0
	Adenocarcinoma of the cervix	23	23	39.1	17.4	13.0	30.4
	Endometrioid endometrial carcinoma	338	276	15.2	21.0	29.7	34.1
	Endometrial serous carcinoma	86	69	18.8	7.2	15.9	58.0
	Carcinosarcoma of the uterus	57	45	37.8	13.3	8.9	40.0
	Endometrial carcinoma, high grade, G3	13	7	57.1	0.0	14.3	28.6
	Endometrial clear cell carcinoma	9	6	0.0	0.0	0.0	100.0
	Endometrioid carcinoma of the ovary	130	112	16.1	11.6	20.5	51.8
	Serous carcinoma of the ovary	580	480	3.5	5.8	15.0	75.6
	Mucinous carcinoma of the ovary	101	77	64.9	11.7	11.7	11.7
	Clear cell carcinoma of the ovary	51	30	3.3	0.0	0.0	96.7
	Carcinosarcoma of the ovary	47	45	31.1	6.7	2.2	60.0
	Granulosa cell tumor of the ovary	44	42	97.6	0.0	2.4	0.0
	Leydig cell tumor of the ovary	4	4	100.0	0.0	0.0	0.0
	Sertoli cell tumor of the ovary	1	1	100.0	0.0	0.0	0.0
	Sertoli Leydig cell tumor of the ovary	3	3	100.0	0.0	0.0	0.0
	Steroid cell tumor of the ovary	3	3	100.0	0.0	0.0	0.0
	Brenner tumor	41	41	95.1	0.0	2.4	2.4
Tumors of the breast	Invasive breast carcinoma of no special type	499	495	100.0	0.0	0.0	0.0
	Lobular carcinoma of the breast	192	184	100.0	0.0	0.0	0.0
	Medullary carcinoma of the breast	23	20	100.0	0.0	0.0	0.0
	Tubular carcinoma of the breast	20	14	100.0	0.0	0.0	0.0
	Mucinous carcinoma of the breast	29	28	100.0	0.0	0.0	0.0
	Phyllodes tumor of the breast	50	48	100.0	0.0	0.0	0.0
Tumors of the digestive system	Adenomatous polyp, low-grade dysplasia	50	48	100.0	0.0	0.0	0.0
	Adenomatous polyp, high-grade dysplasia	50	49	100.0	0.0	0.0	0.0
	Adenocarcinoma of the colon	2483	2270	99.9	0.0	0.0	0.1
	Gastric adenocarcinoma, diffuse type	215	186	100.0	0.0	0.0	0.0
	Gastric adenocarcinoma, intestinal type	215	193	100.0	0.0	0.0	0.0
	Gastric adenocarcinoma, mixed type	62	60	100.0	0.0	0.0	0.0
	Adenocarcinoma of the esophagus	83	66	100.0	0.0	0.0	0.0
	Squamous cell carcinoma of the esophagus	76	55	100.0	0.0	0.0	0.0
	Squamous cell carcinoma of the anal canal	91	84	100.0	0.0	0.0	0.0
	Cholangiocarcinoma	121	115	100.0	0.0	0.0	0.0
	Gallbladder adenocarcinoma	51	45	100.0	0.0	0.0	0.0
	Gallbladder Klatskin tumor	42	41	100.0	0.0	0.0	0.0
	Hepatocellular carcinoma	312	310	100.0	0.0	0.0	0.0
	Ductal adenocarcinoma of the pancreas	659	616	99.2	0.2	0.5	0.2
	Pancreatic/ampullary adenocarcinoma	98	94	100.0	0.0	0.0	0.0
	Acinar cell carcinoma of the pancreas	18	18	100.0	0.0	0.0	0.0
	Gastrointestinal stromal tumor (GIST)	62	58	100.0	0.0	0.0	0.0
	Appendix, neuroendocrine tumor (NET)	25	16	100.0	0.0	0.0	0.0
	Colorectal, neuroendocrine tumor (NET)	12	11	100.0	0.0	0.0	0.0
	Ileum, neuroendocrine tumor (NET)	53	49	100.0	0.0	0.0	0.0
	Pancreas, neuroendocrine tumor (NET)	101	92	100.0	0.0	0.0	0.0
	Colorectal, neuroendocrine carcinoma (NEC)	14	12	100.0	0.0	0.0	0.0
	Ileum, neuroendocrine carcinoma (NEC)	8	8	100.0	0.0	0.0	0.0
	Gallbladder, neuroendocrine carcinoma (NEC)	4	3	100.0	0.0	0.0	0.0
	Pancreas, neuroendocrine carcinoma (NEC)	14	14	100.0	0.0	0.0	0.0
Tumors of the urinary system	Non-invasive papillary urothelial ca., pTa G2 low grade	177	152	76.3	9.2	9.9	4.6
	Non-invasive papillary urothelial ca., pTa G2 high grade	141	116	87.1	1.7	6.9	4.3
	Non-invasive papillary urothelial carcinoma, pTa G3	219	128	97.7	0.8	0.8	0.8
	Urothelial carcinoma, pT2-4 G3	735	597	97.5	1.0	0.7	0.8
	Squamous cell carcinoma of the bladder	22	22	100.0	0.0	0.0	0.0
	Small cell neuroendocrine carcinoma of the bladder	5	5	100.0	0.0	0.0	0.0
	Sarcomatoid urothelial carcinoma	25	23	91.3	4.3	0.0	4.3
	Urothelial carcinoma of the kidney pelvis	62	60	85.0	10.0	5.0	0.0
	Clear cell renal cell carcinoma	1286	1188	16.5	8.7	27.9	47.0
	Papillary renal cell carcinoma	368	317	2.2	6.6	13.9	77.3
	Clear cell (tubulo) papillary renal cell tumor	26	23	17.4	0.0	13.0	69.6
	Chromophobe renal cell carcinoma	170	145	10.3	3.4	33.1	53.1
	Oncocytoma	257	226	3.1	6.6	30.1	60.2
Tumors of the male genital organs	Adenocarcinoma of the prostate, Gleason 3 + 3	83	79	100.0	0.0	0.0	0.0
	Adenocarcinoma of the prostate, Gleason 4 + 4	80	70	100.0	0.0	0.0	0.0
	Adenocarcinoma of the prostate, Gleason 5 + 5	85	79	100.0	0.0	0.0	0.0
	Adenocarcinoma of the prostate (recurrence)	258	216	100.0	0.0	0.0	0.0
	Small cell neuroendocrine carcinoma of the prostate	2	2	100.0	0.0	0.0	0.0
	Seminoma	682	573	100.0	0.0	0.0	0.0
	Embryonal carcinoma of the testis	54	41	100.0	0.0	0.0	0.0
	Leydig cell tumor of the testis	31	31	100.0	0.0	0.0	0.0
	Sertoli cell tumor of the testis	2	2	100.0	0.0	0.0	0.0
	Sex cord stromal tumor of the testis	1	1	100.0	0.0	0.0	0.0
	Spermatocytic tumor of the testis	1	1	100.0	0.0	0.0	0.0
	Yolk sac tumor	53	45	100.0	0.0	0.0	0.0
	Teratoma	53	34	97.1	0.0	0.0	2.9
	Squamous cell carcinoma of the penis	92	90	100.0	0.0	0.0	0.0
Tumors of endocrine organs	Adenoma of the thyroid gland	113	106	0.0	0.9	2.8	96.2
	Papillary thyroid carcinoma	391	370	1.4	1.4	7.3	90.0
	Follicular thyroid carcinoma	154	143	0.7	1.4	4.9	93.0
	Medullary thyroid carcinoma	111	95	100.0	0.0	0.0	0.0
	Parathyroid gland adenoma	43	42	100.0	0.0	0.0	0.0
	Anaplastic thyroid carcinoma	45	42	59.5	11.9	9.5	19.0
	Adrenal cortical adenoma	50	36	97.2	0.0	0.0	2.8
	Adrenal cortical carcinoma	28	28	100.0	0.0	0.0	0.0
	Phaeochromocytoma	50	50	100.0	0.0	0.0	0.0
Tumors of hemotopoetic and lymphoid tissues	Hodgkin Lymphoma	103	89	100.0	0.0	0.0	0.0
	Small lymphocytic lymphoma, B-cell type	50	50	100.0	0.0	0.0	0.0
	Diffuse large B cell lymphoma (DLBCL)	113	113	100.0	0.0	0.0	0.0
	Follicular lymphoma	88	88	100.0	0.0	0.0	0.0
	T-cell non-Hodgkin lymphoma	25	25	100.0	0.0	0.0	0.0
	Mantle cell lymphoma	18	18	100.0	0.0	0.0	0.0
	Marginal zone lymphoma	16	16	100.0	0.0	0.0	0.0
	Diffuse large B-cell lymphoma (DLBCL) in the testis	16	16	93.8	6.3	0.0	0.0
	Burkitt lymphoma	5	5	100.0	0.0	0.0	0.0
Tumors of soft tissue and bone	Granular cell tumor	23	20	100.0	0.0	0.0	0.0
	Leiomyoma	50	45	100.0	0.0	0.0	0.0
	Leiomyosarcoma	94	87	100.0	0.0	0.0	0.0
	Liposarcoma	96	91	100.0	0.0	0.0	0.0
	Malignant peripheral nerve sheath tumor (MPNST)	15	14	100.0	0.0	0.0	0.0
	Myofibrosarcoma	26	26	100.0	0.0	0.0	0.0
	Angiosarcoma	42	40	100.0	0.0	0.0	0.0
	Angiomyolipoma	91	89	100.0	0.0	0.0	0.0
	Dermatofibrosarcoma protuberans	21	16	100.0	0.0	0.0	0.0
	Ganglioneuroma	14	13	100.0	0.0	0.0	0.0
	Kaposi sarcoma	8	5	100.0	0.0	0.0	0.0
	Neurofibroma	117	115	100.0	0.0	0.0	0.0
	Sarcoma, not otherwise specified (NOS)	74	69	97.1	2.9	0.0	0.0
	Paraganglioma	41	40	100.0	0.0	0.0	0.0
	Ewing sarcoma	23	18	100.0	0.0	0.0	0.0
	Rhabdomyosarcoma	7	7	100.0	0.0	0.0	0.0
	Schwannoma	122	119	100.0	0.0	0.0	0.0
	Synovial sarcoma	12	11	100.0	0.0	0.0	0.0
	Osteosarcoma	19	19	100.0	0.0	0.0	0.0
	Chondrosarcoma	15	10	100.0	0.0	0.0	0.0
	Rhabdoid tumor	5	5	100.0	0.0	0.0	0.0
	Solitary fibrous tumor	17	17	94.1	0.0	0.0	5.9

**Table 2:** PAX8 immunostaining and tumor phenotype

		*n*	PAX8 immunostaining				*p*
			Negative (%)	Weak (%)	Moderate (%)	Strong (%)	
Clear cell renal cell carcinoma	ISUP 1	268	21.6	9.3	26.1	42.9	0.4906
	ISUP 2	401	18.7	8.2	27.4	45.6	
	ISUP 3	266	13.2	10.9	30.1	45.9	
	ISUP 4	73	16.4	8.2	27.4	47.9	
	Fuhrman 1	64.0	9.4	6.3	25.0	59.4	0.3200
	Fuhrman 2	683	19.0	8.1	27.1	45.8	
	Fuhrman 3	295	13.6	10.2	28.5	47.8	
	Fuhrman 4	88	17.0	8.0	27.3	47.7	
	Thoenes 1	352	18.8	8.5	26.1	46.6	0.8217
	Thoenes 2	492	18.9	10.2	28.0	42.9	
	Thoenes 3	98	15.3	8.2	26.5	50.0	
	UICC 1	337	15.7	9.2	23.4	51.6	0.0368
	UICC 2	38	21.1	15.8	26.3	36.8	
	UICC 3	92	15.2	13.0	33.7	38.0	
	UICC 4	75	26.7	14.7	26.7	32.0	
	pT1	682	14.5	7.8	27.4	50.3	0.0411
	pT2	131	22.1	9.9	31.3	36.6	
	pT3-4	323	19.5	9.9	25.7	44.9	
	pN0	173	16.8	12.1	26.0	45.1	0.5232
	pN +	25	16.0	4.0	24.0	56.0	
	pM0	113	13.3	13.3	27.4	46.0	0.2713
	pM +	92	23.9	12.0	25.0	39.1	
Urothelial bladder carcinoma	pTa G2 low	152	76.3	9.2	9.9	4.6	< 0.0001
	pTa G2 high	116	87.1	1.7	6.9	4.3	
	pTa G3	97	100.0	0.0	0.0	0.0	
	pT2	130	99.2	0.0	0.0	0.8	0.2805*
	pT3	213	97.2	1.4	0.5	0.9	
	pT4	98	94.9	2.0	2.0	1.0	
	G2	23	87.0	0.0	4.3	8.7	0.0224*
	G3	417	97.8	1.2	0.5	0.5	
	pN0	257	98.1	0.4	0.4	1.2	0.3199*
	pN +	162	96.3	1.9	1.2	0.6	
Endometrioid endometrial carcinoma	pT1	87	12.6	18.4	31.0	37.9	0.5813
	pT2	19	21.1	15.8	47.4	15.8	
	pT3-4	24	12.5	20.8	33.3	33.3	
	pN0	35	11.4	20.0	37.1	31.4	0.9258
	pN +	20	15.0	25.0	30.0	30.0	
Serous carcinoma of the ovary	pT1	28	0.0	0.0	21.4	78.6	0.2090
	pT2	40	2.5	2.5	12.5	82.5	
	pT3	229	3.1	7.9	16.2	72.9	
	pN0	68	1.5	2.9	20.6	75.0	0.2378
	pN1	148	4.1	8.1	14.9	73.0	

*Only in pT2-4 urothelial bladder carcinomas, abbreviation: *pT* pathological tumor stage, *G* grade, *pN* pathological lymph node status, *pM* pathological status of distant metastasis, *ISUP* International Society of Urological Pathology, *UICC* Union for International Cancer Control

### Comparison of PAX8 antibodies

A subset of 1,009 tumor samples from 23 different tumor types was analyzed with both MSVA-708R and MRQ-50. Both antibodies stained comparably positive in 472 ovarian cancers, including 98.1% (MSVA-708R) and 97.2% (MRQ-50) of serous, 95.7% and 100% of clear cell carcinomas, and 62.5% each of ovarian carcinosarcomas (supplementary Fig. 2). MSVA-708R had a higher sensitivity (63.6%) as compared to MRQ-50 (33.3%) in mucinous and endometrioid (90.9% vs. 78.8%) ovarian cancers. A concordantly negative result was found in 42 tumors including acinar cell carcinomas of the pancreas and neuroendocrine tumors of the lung, colorectum, and appendix. However, exclusive staining with MRQ-50 (but not with MSVA-708R) was found in 44.4% of 171 neuroendocrine neoplasias of various origin and in 87.6% of 275 lymphomas (supplementary Fig. 3). All data are summarized in supplementary Fig. 4.

### Sensitivity and specificity calculations

**Fig. 5:**
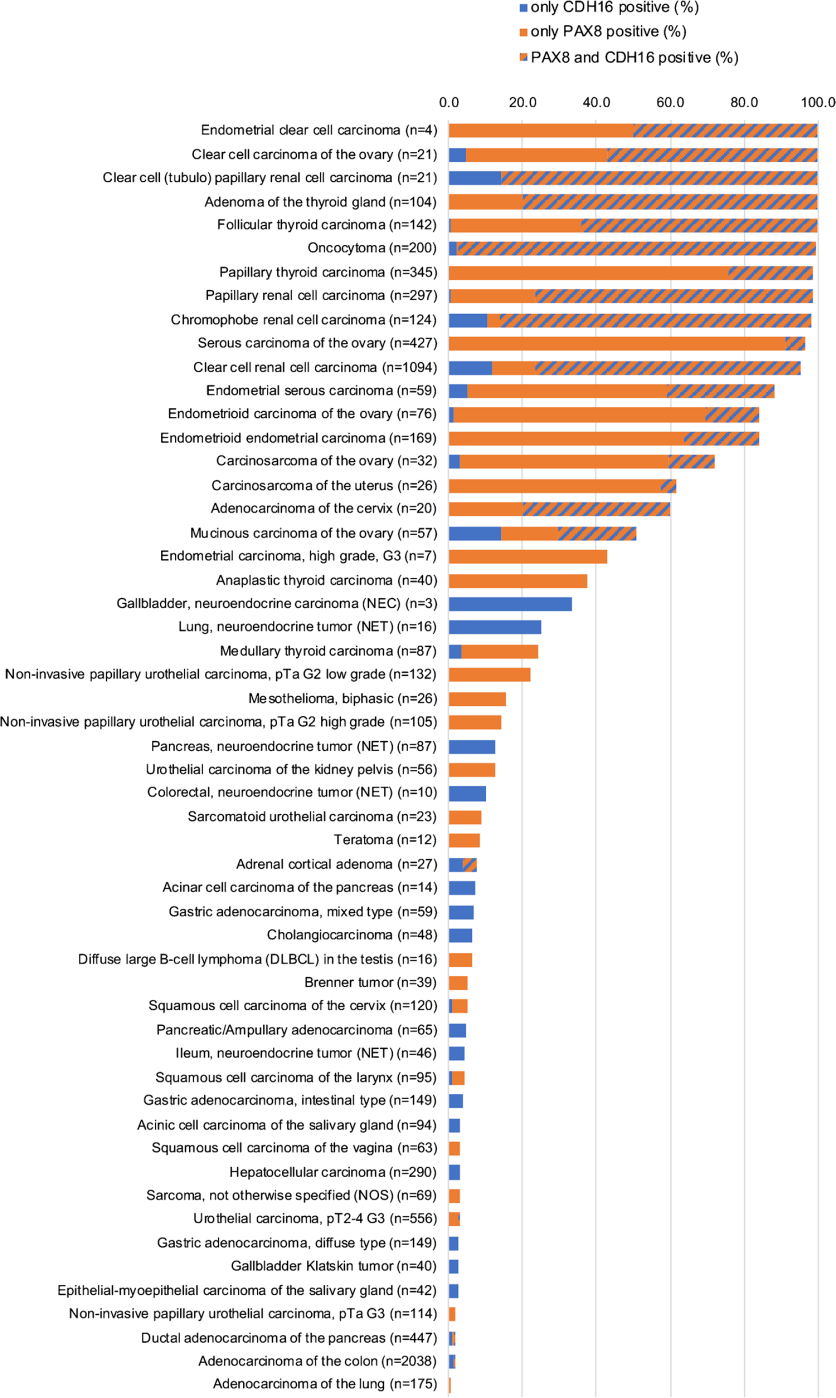
PAX8 and CDH16 immunostaining in human tumors

Data on the expression of CDH16, GATA3, and p63 were available from subsets of the tumors for which PAX8 data were collected in our project. Results from a comparative analysis of PAX8 and CDH16 are shown in Fig. 5 and supplementary Table 1. These data show that PAX8/CDH16 dual positivity almost exclusively occurred in neoplasms derived from kidney, thyroid, uterus, and ovary. Various further tumor entities showed positive staining either for PAX8 or CDH16 but not for both. For the distinction between a renal cell origin and a non-renal origin of tumors (including all other tumor entities of our study), sensitivity was 88.1% and specificity 87.2% for PAX8, while sensitivity was 85.3% and specificity 95.7% for CDH16. The combination of PAX8 and CDH16 increased specificity to 96.6%. For the distinction between renal cell carcinomas and urothelial carcinomas, sensitivity was 86.7% and specificity 91.3% for PAX8, while sensitivity was 82.7% and specificity 99.8% for CDH16. The combination of PAX8 and CDH16 increased specificity to 99.9%. For comparison with established markers for the distinction between urothelial carcinomas and renal cell carcinomas, we also performed sensitivity and specificity calculation of p63 and GATA3. Sensitivity was 86.5% and specificity 100% for p63, while sensitivity was 83.7% and specificity 98.3% for GATA3. All data are summarized in Table 3.

**Table 3:** Sensitivity and specificity of PAX8 and CDH16 to determine renal tumor origin

Purpose	Marker	Sensitivity	Specificity
Distinction between renal cell origin and all other tumor entities of this study	PAX8	0.881	0.872
	CDH16	0.853	0.957
	PAX8 and CDH16	0.766	0.966
	PAX8 and/or CDH16	0.968	0.862
Distinction between renal cell carcinoma and urothelial carcinoma	PAX8	0.867	0.931
	CDH16	0.827	0.998
	PAX8 and CDH16	0.739	0.999
	PAX8 and/or CDH16	0.964	0.932
Distinction between urothelial carcinoma and renal cell carcinoma	p63	0.865	1.000
	GATA3	0.837	0.983
	P63 and GATA3	0.807	1.000
	P63 and/or GATA3	0.977	0.985

## Discussion

The successful analysis of 15,223 cancers provides a comprehensive overview on PAX8 expression in cancer. That PAX8 positivity was most commonly seen in neoplasms of the thyroid (follicular), the kidney, and the female genital tract was expected based on numerous earlier studies describing high PAX8 positivity rates in these entities [20, 30, 43] and because the pattern of protein expression of tumor cells typically reflects the expression of corresponding normal cells. These results also support the previously suggested utility of PAX8 for the distinction of these tumor entitles from other cancer types especially in case of metastatic disease (summarized in [39]). This is all the more true since other important tumor entities that often metastasize were almost always PAX8 negative. Tumors with close to 100% PAX8 negativity for example included carcinomas of the breast and the liver, gastric, prostatic, pancreatic, and pulmonary adenocarcinomas, as well as small cell carcinomas and neuroendocrine tumors of various sites. For most of these PAX8-negative tumor types, several other studies have described significant PAX8 positivity rates often exceeding 20% [11, 34, 40, 51] or even 50% [16, 20, 22, 43]. Antibody cross-reactivities for PAX5 and/or PAX6 which are known to occur with monoclonal [22] and polyclonal PAX8 antibodies [48] might represent a major cause for these discrepancies.

To avoid an impact of antibody cross-reactivity on our data, emphasis was placed on a thorough validation of our assay. The International Working Group for Antibody Validation (IWGAV) has suggested that either a comparison of the findings obtained by two different independent antibodies or a comparison with expression data obtained by another independent method should be performed to validate antibodies for IHC on formalin-fixed tissues [50]. As an independent approach for expression measurement, compiled RNA data from three independent RNA screening studies were used [7, 23, 24, 45]. These projects had identified PAX8 RNA only in kidney, epididymis, seminal vesicle, endometrium, cervix uteri, fallopian tube, and thyroid. The fact hat the  immunohistochemical PAX8 analysis of 76 different normal tissue categories by MSVA-708R revealed nuclear positivity in only these organs supports the validity of our assay. True expression of PAX8 in all cell types with a nuclear PAX8 positivity is further validated by the confirmation of all these stainings by our second anti-PAX8 antibody MRQ-50. The use of a very broad range of normal tissues for antibody validation increases the likelihood for detecting undesired cross-reactivities because virtually all proteins occurring in normal cells of adult humans are subjected to the validation experiment. Additional nuclear staining of lymphocytes, thymic epithelial cells, the parathyroid, and several neuroendocrine cell types that were only observed by MRQ-50 were therefore considered specific cross-reactivities of this antibody. Accordingly, a considerable fraction of neuroendocrine neoplasms and lymphomas stained positive for MRQ-50 and not for MSVA-708R, although the positivity rates of these antibodies were comparable in ovarian tumors. Cytoplasmic staining of few inflammatory cells of the intestine, a subset of cells of the adenohypophysis and of gastric glands which were not seen by MRQ-50, was identified as cross-reactivities of MSVA-708R. These, however, do not cause interpretation issues as they are non-nuclear.

The large scale of our study enabled a ranking list of human tumor entities according to the prevalence of positive PAX8 immunostaining. As it is evident from the summarized literature (Supplementary Fig. 2), this list could not have been easily compiled from the published literature because of the high diversity of published data. The use of TMAs did not only enable the scale of the study but also allowed for a very high level of standardization, which not only included traditional experimental factors such as incubation time, temperature, and antibody concentration but also other important factors such as section age and the quantity of tumor analyzed per patient. Section age of 2 weeks and higher already results in a significant reduction of staining for many antibodies [17, 27]. The fact that the tissue quantity of tumor analyzed affects the positivity rate is already seen in studies comparing one, two, or three cores per tissue block on a TMA [4, 14, 37]. Of note, the only study comparing IHC findings obtained from large sections versus TMAs containing one or several tissue cores found almost twice as many p53 positive cases on whole sections than on TMAs, but the prognostic role of p53 positivity was only found for TMA and not for whole section data [47]. While these data show that the use of larger tissue quantities also increases the risk for finding artificial or irrelevant staining, they emphasize the desirability for standardizing tissue quantities for comparative tumor tissue analysis. The quantity of tissue on a TMA spot is not much different from the amount of tumor which is often contained in small biopsies. It must be considered, however, that some false negative cases always occur in TMA studies due to preanalytical tissue damage.

Previously published PAX8 IHC data for different tumor types are shown in comparison to our results in supplementary Fig. 5 and supplementary Table 2. Based on our comparison of MSVA-708R and MRQ-50, we assume that very high positivity rates in many tumor entities that were largely PAX8 negative in our cohort were driven by similar antibody-specific cross-reactivities. These especially include B-cell lymphomas [31], neoplasms of the thymus [42], neuroendocrine tumors [22], and Merkel cell carcinomas [38]. The PAX8 positivity rates of 0–75% reported earlier for medullary carcinomas of the thyroid may to some extent be caused by PAX8 antibodies cross-reacting with PAX6 [13, 29, 48]. In addition, interspersed normal follicular cells which are commonly seen between tumor cell layers (Fig. 2H) may have contributed to the perception of PAX8 positivity in medullary cancers [13]. It is also of note that we had seen PAX8 positivity neither in 4 samples of normal parathyroid nor in 43 parathyroidal adenomas by MSVA-708R, while normal parathyroid was stained strongly by MRQ-50. Altinay et al. [1] had recently suggested that PAX8 analysis by MRQ-50 may distinguish parathyroidal adenoma (positive in 85%) from normal parathyroid (100% negative).

That PAX8 positivity was also seen in 88 cancers from 118 tumor entities that were not derived from thyroid, kidney, or the female genital tract represents a diagnostic challenge. Considering that urothelial neoplasms are among these occasionally PAX8-positive tumors, PAX8 cannot reliably be used to distinguish urothelial carcinoma from renal cancer in kidney masses [2] or from ovarian or endometrial cancer in pelvic masses as previously suggested [52]. In an earlier comparative study, our group had identified CDH16 as a suitable marker for the distinction of renal cell carcinomas from other tumor entities [21]. The combined analysis of PAX8 and CDH16 data suggests that this combination could be useful. Almost all of the 1714 (99.9%) tumors with PAX8/CDH16 dual positivity were derived from kidney/female genital tract/thyroid. Of note, CDH16 is not a recommendable marker for thyroid cancer detection, because it is often lost in thyroidal carcinomas.

Sporadic reports on a possible prognostic impact of PAX8 in different tumor types [3, 5, 6, 15, 26, 44] and the availability of a clinical database to our TMAs prompted us to search for possible associations between PAX8 expression and cancer aggressiveness. The evaluation of large cohorts of urinary bladder and renal cell carcinomas, as well as ovarian and endometrial cancers, did not suggest a major clinical and prognostic impact of PAX8 expression in these entities. The significant association between high PAX8 expression and low tumor grade in non-invasive (pTa) urinary bladder cancers fits well to the weak to moderate nuclear PAX8 staining that was occasionally seen in normal urothelium, which appears to be lost during tumor progression.

In summary, the standardized assessment of PAX8 staining in 149 different tumor types and subtypes enabled us to define a ranking order with respect to the frequency of PAX8 immunostaining in tumors. Although neoplasms derived from kidney, thyroid, and the female genital tract were most often and most strongly PAX8 positive, there were 15 additional tumor types exhibiting PAX8 positivity at least in occasional cases. A parallel analysis of CDH16 may efficiently complement PAX8 IHC to discriminate tumors from the kidney, the female genital tract, or the thyroid.

## Supplementary Information

Below is the link to the electronic supplementary material.

Supplementary file1 (Immunohistochemistry (IHC) validation by comparison of antibodies. The panels show a comparison of IHC results obtained by two independent PAX8 antibodies (MSVA-708R, MRQ-50). Using MSVA-708R, a nuclear PAX8 positivity of variable intensity was seen in distinct cell types of the thyroid (A), kidney (B), seminal vesicle (C), caput epididymis (D), endocervix (E), endometrium (F) and the fallopian tube (G) while staining was absent in parathyroid (H), lymph node (I), pancreas (K), and thymus (L). A purely cytoplasmic staining was seen by MSVA-708R in some inflammatory cells of the gastrointestinal mucosa (M, N) and in some epithelial cells of the adenohypophysis (O). Using clone MRQ-50, a nuclear staining of identical cell types was seen in thyroid (a), kidney (b), seminal vesicle (c), epididymis (d), endocervix (e), endometrium (f) and the fallopian tube (g). In addition, MRQ-50 showed a nuclear staining of parathyroidal epithelial cells (h), a subset of lymphocytes in the lymph node (i), islet cells of the pancreas (and a granular cytoplasmic staining of acinar cells; k), lymphocytes and a subset of epithelial cells of the thymus (l), and of neuroendocrine cells in the gastrointestinal mucosa (m, n) while staining was absent in the adenohypophysis (o). (PDF 1643 KB)

Supplementary file2 (Comparison of anti-PAX8 antibody clones MSVA-708R (left hand side) and MRQ-50 (right hand side) in cancer tissues: Examples of concordant staining. Strong nuclear PAX8 staining is found with both antibodies at comparable frequencies in endometrioid (a,b), serous (c,d), and mucinous ovarian carcinomas (e,f) as well as in carcinosarcomas of the ovaries (g,h). (PDF 506 KB)

Supplementary file3 (Comparison of anti-PAX8 antibody clones MSVA-708R (left hand side) and MRQ-50 (right hand side) in cancer tissues: Examples of discordant staining. Strong nuclear staining with MRQ-50 in a neuroendocrine tumor of the pancreas (b), in neuroendocrine carcinomas of the colon (d), and gallbladder (f) and in a follicular lymphoma (h). These tumor types stain entirely negative with MSVA-708R (a, c, e, g). (PDF 504 KB)

Supplementary file4 (Comparison of PAX8 antibodies in 23 tumor types. (PDF 16 KB)

Supplementary file5 (Comparison with previous PAX8 literature. An „X“ indicates the fraction of PAX8 positive cancers in the present study, dots indicate the reported frequencies from the literature for comparison: red dots mark studies with ≤ 10 analyzed tumors, yellow dots mark studies with 11 to 25 analyzed tumors, and green dots mark studies with > 25 analyzed tumors. References are found in supplementary table 2. (PDF 49 KB)

Supplementary file6 (PAX8 and CDH16 immunostaining in human tumors. (XLSX 18 KB)

Supplementary file7 (Previous PAX8 immunohistochemistry studies. (XLSX 30 KB)

## Data Availability

All data generated or analyzed during this study are included in this published article.

## References

[1] Altinay S, Erozgur B, Dural AC, Volante M, Papotti MG (2021) Monoclonal/polyclonal PAX-8, PTH and GATA3 immunohistochemistry in parathyroid lesions. J Endocrinol Invest 44:1997–2008. 10.1007/s40618-021-01518-3 33566340 10.1007/s40618-021-01518-3

[2] Barr ML, Jilaveanu LB, Camp RL, Adeniran AJ, Kluger HM, Shuch B (2015) PAX-8 expression in renal tumours and distant sites: a useful marker of primary and metastatic renal cell carcinoma? J Clin Pathol 68:12–17. 10.1136/jclinpath-2014-202259 25315900 10.1136/jclinpath-2014-202259 PMC4429054

[3] Becker N, Chernock RD, Nussenbaum B, Lewis JS Jr (2014) Prognostic significance of beta-human chorionic gonadotropin and PAX8 expression in anaplastic thyroid carcinoma. Thyroid 24:319–326. 10.1089/thy.2013.0117 23806007 10.1089/thy.2013.0117

[4] Camp RL, Charette LA, Rimm DL (2000) Validation of tissue microarray technology in breast carcinoma. Lab Invest 80:1943–1949. 10.1038/labinvest.3780204 11140706 10.1038/labinvest.3780204

[5] Chai HJ, Ren Q, Fan Q, Ye L, Du GY, Du HW, Xu W, Li Y, Zhang L, Cheng ZP (2017) PAX8 is a potential marker for the diagnosis of primary epithelial ovarian cancer. Oncol Lett 14:5871–5875. 10.3892/ol.2017.6949 29113220 10.3892/ol.2017.6949 PMC5661437

[6] Chelariu-Raicu A, Holley E, Mayr D, Klauschen F, Wehweck F, Rottmann M, Kessler M, Kaltofen T, Czogalla B, Trillsch F, Mahner S, Schmoeckel E (2022) A combination of immunohistochemical markers, MUC1, MUC5AC, PAX8 and growth pattern for characterization of mucinous neoplasm of the ovary. Int J Gynecol Cancer 32:662–668. 10.1136/ijgc-2021-003104 35185017 10.1136/ijgc-2021-003104

[7] Consortium GT (2013) The genotype-tissue expression (GTEx) project. Nat Genet 45:580–585. 10.1038/ng.2653 23715323 10.1038/ng.2653 PMC4010069

[8] Dancau AM, Simon R, Mirlacher M, Sauter G (2016) Tissue microarrays methods Mol Biol 1381:53–65. 10.1007/978-1-4939-3204-7_3 26667454 10.1007/978-1-4939-3204-7_3

[9] De Felice M, Di Lauro R (2004) Thyroid development and its disorders: genetics and molecular mechanisms. Endocr Rev 25:722–746. 10.1210/er.2003-0028 15466939 10.1210/er.2003-0028

[10] Fraizer GC, Shimamura R, Zhang X, Saunders GF (1997) PAX 8 regulates human WT1 transcription through a novel DNA binding site. J Biol Chem 272:30678–30687. 10.1074/jbc.272.49.30678 9388203 10.1074/jbc.272.49.30678

[11] Gailey MP, Bellizzi AM (2013) Immunohistochemistry for the novel markers glypican 3, PAX8, and p40 (DeltaNp63) in squamous cell and urothelial carcinoma. Am J Clin Pathol 140:872–880. 10.1309/AJCP4NSKW5TLGTDS 24225756 10.1309/AJCP4NSKW5TLGTDS

[12] Goyal A, Masand RP, Roma AA (2016) Value of PAX-8 and SF-1 immunohistochemistry in the distinction between female adnexal tumor of probable wolffian origin and its mimics. Int J Gynecol Pathol 35:167–175. 10.1097/PGP.0000000000000222 26352548 10.1097/PGP.0000000000000222

[13] Gucer H, Caliskan S, Kefeli M, Mete O (2020) Do you know the details of your PAX8 antibody? Monoclonal PAX8 (MRQ-50) is not expressed in a series of 45 medullary thyroid carcinomas. Endocr Pathol 31:33–38. 10.1007/s12022-019-09603-3 31912298 10.1007/s12022-019-09603-3

[14] Hoos A, Urist MJ, Stojadinovic A, Mastorides S, Dudas ME, Leung DH, Kuo D, Brennan MF, Lewis JJ, Cordon-Cardo C (2001) Validation of tissue microarrays for immunohistochemical profiling of cancer specimens using the example of human fibroblastic tumors. Am J Pathol 158:1245–1251. 10.1016/S0002-9440(10)64075-8 11290542 10.1016/S0002-9440(10)64075-8 PMC1891917

[15] Hu S, Gan H, Yang F (2022) Significance analysis of PAX8 expression in endometrial carcinoma. Medicine (Baltimore) 101:e31159. 10.1097/MD.0000000000031159 36281161 10.1097/MD.0000000000031159 PMC9592497

[16] Hu A, Li H, Zhang L, Ren C, Wang Y, Liu Y, Liu C (2015) Differentiating primary and extragenital metastatic mucinous ovarian tumours: an algorithm combining PAX8 with tumour size and laterality. J Clin Pathol 68:522–528. 10.1136/jclinpath-2015-202951 25827135 10.1136/jclinpath-2015-202951 PMC4484043

[17] Jacobs TW, Prioleau JE, Stillman IE, Schnitt SJ (1996) Loss of tumor marker-immunostaining intensity on stored paraffin slides of breast cancer. J Natl Cancer Inst 88:1054–1059. 10.1093/jnci/88.15.1054 8683636 10.1093/jnci/88.15.1054

[18] Kobel M, Kalloger SE, Boyd N, McKinney S, Mehl E, Palmer C, Leung S, Bowen NJ, Ionescu DN, Rajput A, Prentice LM, Miller D, Santos J, Swenerton K, Gilks CB, Huntsman D (2008) Ovarian carcinoma subtypes are different diseases: implications for biomarker studies. PLoS Med 5:e232. 10.1371/journal.pmed.0050232 19053170 10.1371/journal.pmed.0050232 PMC2592352

[19] Kononen J, Bubendorf L, Kallioniemi A, Barlund M, Schraml P, Leighton S, Torhorst J, Mihatsch MJ, Sauter G, Kallioniemi OP (1998) Tissue microarrays for high-throughput molecular profiling of tumor specimens. Nat Med 4:844–847. 10.1038/nm0798-844 9662379 10.1038/nm0798-844

[20] Laury AR, Perets R, Piao H, Krane JF, Barletta JA, French C, Chirieac LR, Lis R, Loda M, Hornick JL, Drapkin R, Hirsch MS (2011) A comprehensive analysis of PAX8 expression in human epithelial tumors. Am J Surg Pathol 35:816–826. 10.1097/PAS.0b013e318216c112 21552115 10.1097/PAS.0b013e318216c112

[21] Lennartz M, Csomos H, Chirico V, Weidemann S, Gorbokon N, Menz A, Buscheck F, Hube-Magg C, Hoflmayer D, Bernreuther C, Blessin NC, Lebok P, Sauter G, Steurer S, Burandt E, Dum D, Krech T, Simon R, Minner S, Jacobsen F, Clauditz TS, Luebke AM, Siraj AK, Al-Dayel F, Al-Kuraya KS, Hinsch A (2023) Cadherin-16 (CDH16) immunohistochemistry: a useful diagnostic tool for renal cell carcinoma and papillary carcinomas of the thyroid. Sci Rep 13:12917. 10.1038/s41598-023-39945-2 37558687 10.1038/s41598-023-39945-2 PMC10412623

[22] Liau JY, Tsai JH, Jeng YM, Kuo KT, Huang HY, Liang CW, Yang CY (2016) The diagnostic utility of PAX8 for neuroendocrine tumors: an immunohistochemical reappraisal Appl Immunohistochem. Mol Morphol 24:57–63. 10.1097/PAI.0000000000000149 10.1097/PAI.0000000000000149 25710581

[23] Lizio M, Abugessaisa I, Noguchi S, Kondo A, Hasegawa A, Hon CC, de Hoon M, Severin J, Oki S, Hayashizaki Y, Carninci P, Kasukawa T, Kawaji H (2019) Update of the FANTOM web resource: expansion to provide additional transcriptome atlases. Nucleic Acids Res 47:D752–D758. 10.1093/nar/gky1099 30407557 10.1093/nar/gky1099 PMC6323950

[24] Lizio M, Harshbarger J, Shimoji H, Severin J, Kasukawa T, Sahin S, Abugessaisa I, Fukuda S, Hori F, Ishikawa-Kato S, Mungall CJ, Arner E, Baillie JK, Bertin N, Bono H, de Hoon M, Diehl AD, Dimont E, Freeman TC, Fujieda K, Hide W, Kaliyaperumal R, Katayama T, Lassmann T, Meehan TF, Nishikata K, Ono H, Rehli M, Sandelin A, Schultes EA, t Hoen PA, Tatum Z, Thompson M, Toyoda T, Wright DW, Daub CO, Itoh M, Carninci P, Hayashizaki Y, Forrest AR, Kawaji H, consortium F, (2015) Gateways to the FANTOM5 promoter level mammalian expression atlas. Genome Biol 16:22. 10.1186/s13059-014-0560-6 25723102 10.1186/s13059-014-0560-6 PMC4310165

[25] Mansouri A, Hallonet M, Gruss P (1996) Pax genes and their roles in cell differentiation and development. Curr Opin Cell Biol 8:851–857 8939674 10.1016/s0955-0674(96)80087-1

[26] Mhawech-Fauceglia P, Wang D, Samrao D, Godoy H, Pejovic T, Liu S, Lele S (2012) Pair-Box (PAX8) protein-positive expression is associated with poor disease outcome in women with endometrial cancer. Br J Cancer 107:370–374. 10.1038/bjc.2012.241 22644304 10.1038/bjc.2012.241 PMC3394976

[27] Mirlacher M, Kasper M, Storz M, Knecht Y, Durmuller U, Simon R, Mihatsch MJ, Sauter G (2004) Influence of slide aging on results of translational research studies using immunohistochemistry. Mod Pathol 17:1414–1420. 10.1038/modpathol.3800208 15205686 10.1038/modpathol.3800208

[28] Narlis M, Grote D, Gaitan Y, Boualia SK, Bouchard M (2007) Pax2 and pax8 regulate branching morphogenesis and nephron differentiation in the developing kidney. J Am Soc Nephrol 18:1121–1129. 10.1681/ASN.2006070739 17314325 10.1681/ASN.2006070739

[29] Nonaka D, Tang Y, Chiriboga L, Rivera M, Ghossein R (2008) Diagnostic utility of thyroid transcription factors Pax8 and TTF-2 (FoxE1) in thyroid epithelial neoplasms. Mod Pathol 21:192–200. 10.1038/modpathol.3801002 18084247 10.1038/modpathol.3801002

[30] Ozcan A, Shen SS, Hamilton C, Anjana K, Coffey D, Krishnan B, Truong LD (2011) PAX 8 expression in non-neoplastic tissues, primary tumors, and metastatic tumors: a comprehensive immunohistochemical study. Mod Pathol 24:751–764. 10.1038/modpathol.2011.3 21317881 10.1038/modpathol.2011.3

[31] Parakh R, Cheng L, Tretiakova M (2018) PAX8-positive B-cell lymphoma in adrenal gland masquerading as metastatic renal cell carcinoma. Int J Surg Pathol 26:721–724. 10.1177/1066896918770022 29665739 10.1177/1066896918770022

[32] Pasca di Magliano M, Di Lauro R, Zannini M (2000) Pax8 has a key role in thyroid cell differentiation. Proc Natl Acad Sci U S A 97:13144–13149. 10.1073/pnas.240336397 11069301 10.1073/pnas.240336397 PMC27192

[33] Pellizzari L, Puppin C, Mariuzzi L, Saro F, Pandolfi M, Di Lauro R, Beltrami CA, Damante G (2006) PAX8 expression in human bladder cancer. Oncol Rep 16:1015–1020 17016586

[34] Pocrnich CE, Ramalingam P, Euscher ED, Malpica A (2016) Neuroendocrine carcinoma of the endometrium: a clinicopathologic study of 25 cases. Am J Surg Pathol 40:577–586. 10.1097/PAS.0000000000000633 26945341 10.1097/PAS.0000000000000633 PMC5998806

[35] Puglisi F, Cesselli D, Damante G, Pellizzari L, Beltrami CA, Di Loreto C (2000) Expression of Pax-8, p53 and bcl-2 in human benign and malignant thyroid diseases. Anticancer Res 20:311–316 10769673

[36] Reiswich V, Schmidt CE, Lennartz M, Hoflmayer D, Hube-Magg C, Weidemann S, Fraune C, Buscheck F, Moller K, Bernreuther C, Simon R, Clauditz TS, Blessin NC, Bady E, Sauter G, Uhlig R, Steurer S, Minner S, Burandt E, Dum D, Marx AH, Krech T, Lebok P, Hinsch A, Jacobsen F (2023) GATA3 expression in human tumors: a tissue microarray study on 16,557 tumors. Pathobiology 90:219–232. 10.1159/000527382 36649695 10.1159/000527382 PMC10937041

[37] Rubin MA, Dunn R, Strawderman M, Pienta KJ (2002) Tissue microarray sampling strategy for prostate cancer biomarker analysis. Am J Surg Pathol 26:312–319. 10.1097/00000478-200203000-00004 11859202 10.1097/00000478-200203000-00004

[38] Sangoi AR, Cassarino DS (2013) PAX-8 expression in primary and metastatic Merkel cell carcinoma: an immunohistochemical analysis. Am J Dermatopathol 35:448–451. 10.1097/DAD.0b013e318271ce53 23612030 10.1097/DAD.0b013e318271ce53

[39] Selves J, Long-Mira E, Mathieu MC, Rochaix P, Ilie M (2018) Immunohistochemistry for diagnosis of metastatic carcinomas of unknown primary site. Cancers (Basel) 10:108. 10.3390/cancers10040108 29621151 10.3390/cancers10040108 PMC5923363

[40] Singh K, Hanley LC, Sung CJ, Quddus MR (2020) Comparison of PAX8 expression in breast carcinoma using MRQ50 and BC12 monoclonal Antibodies. Appl Immunohistochem Mol Morphol 28:558–561. 10.1097/PAI.0000000000000796 31335489 10.1097/PAI.0000000000000796

[41] Steurer S, Riemann C, Buscheck F, Luebke AM, Kluth M, Hube-Magg C, Hinsch A, Hoflmayer D, Weidemann S, Fraune C, Moller K, Menz A, Fisch M, Rink M, Bernreuther C, Lebok P, Clauditz TS, Sauter G, Uhlig R, Wilczak W, Dum D, Simon R, Minner S, Burandt E, Krech R, Krech T, Marx AH (2021) p63 expression in human tumors and normal tissues: a tissue microarray study on 10,200 tumors. Biomark Res 9:7. 10.1186/s40364-021-00260-5 33494829 10.1186/s40364-021-00260-5 PMC7830855

[42] Suzuki A, Hirokawa M, Takada N, Higuchi M, Tanaka A, Hayashi T, Kuma S, Miyauchi A (2018) Utility of monoclonal PAX8 antibody for distinguishing intrathyroid thymic carcinoma from follicular cell-derived thyroid carcinoma. Endocr J 65:1171–1175. 10.1507/endocrj.EJ18-0282 30210064 10.1507/endocrj.EJ18-0282

[43] Tacha D, Zhou D, Cheng L (2011) Expression of PAX8 in normal and neoplastic tissues: a comprehensive immunohistochemical study. Appl Immunohistochem Mol Morphol 19:293–299. 10.1097/PAI.0b013e3182025f66 21285870 10.1097/PAI.0b013e3182025f66

[44] Tao F, Zhu H, Xu J, Guo Y, Wang X, Shao L, Pan D, Li G, Fang R (2024) Prognostic value of PAX8 in small cell lung cancer. Heliyon 10:e28251. 10.1016/j.heliyon.2024.e28251 38596099 10.1016/j.heliyon.2024.e28251 PMC11002052

[45] Thul PJ, Akesson L, Wiking M, Mahdessian D, Geladaki A, Ait Blal H, Alm T, Asplund A, Bjork L, Breckels LM, Backstrom A, Danielsson F, Fagerberg L, Fall J, Gatto L, Gnann C, Hober S, Hjelmare M, Johansson F, Lee S, Lindskog C, Mulder J, Mulvey CM, Nilsson P, Oksvold P, Rockberg J, Schutten R, Schwenk JM, Sivertsson A, Sjostedt E, Skogs M, Stadler C, Sullivan DP, Tegel H, Winsnes C, Zhang C, Zwahlen M, Mardinoglu A, Ponten F, von Feilitzen K, Lilley KS, Uhlen M, Lundberg E (2017) A subcellular map of the human proteome. Science 356:eaal3321. 10.1126/science.aal3321 28495876 10.1126/science.aal3321

[46] Tong GX, Devaraj K, Hamele-Bena D, Yu WM, Turk A, Chen X, Wright JD, Greenebaum E (2011) Pax8: a marker for carcinoma of Mullerian origin in serous effusions. Diagn Cytopathol 39:567–574. 10.1002/dc.21426 20607683 10.1002/dc.21426

[47] Torhorst J, Bucher C, Kononen J, Haas P, Zuber M, Kochli OR, Mross F, Dieterich H, Moch H, Mihatsch M, Kallioniemi OP, Sauter G (2001) Tissue microarrays for rapid linking of molecular changes to clinical endpoints. Am J Pathol 159:2249–2256. 10.1016/S0002-9440(10)63075-1 11733374 10.1016/S0002-9440(10)63075-1 PMC1850582

[48] Toriyama A, Mori T, Sekine S, Yoshida A, Hino O, Tsuta K (2014) Utility of PAX8 mouse monoclonal antibody in the diagnosis of thyroid, thymic, pleural and lung tumours: a comparison with polyclonal PAX8 antibody. Histopathology 65:465–472. 10.1111/his.12405 24592933 10.1111/his.12405

[49] Torres M, Gomez-Pardo E, Dressler GR, Gruss P (1995) Pax-2 controls multiple steps of urogenital development. Development 121:4057–4065 8575306 10.1242/dev.121.12.4057

[50] Uhlen M, Bandrowski A, Carr S, Edwards A, Ellenberg J, Lundberg E, Rimm DL, Rodriguez H, Hiltke T, Snyder M, Yamamoto T (2016) A proposal for validation of antibodies. Nat Methods 13:823–827. 10.1038/nmeth.3995 27595404 10.1038/nmeth.3995 PMC10335836

[51] Weissferdt A, Tang X, Wistuba II, Moran CA (2013) Comparative immunohistochemical analysis of pulmonary and thymic neuroendocrine carcinomas using PAX8 and TTF-1. Mod Pathol 26:1554–1560. 10.1038/modpathol.2013.111 23787439 10.1038/modpathol.2013.111

[52] Xiang L, Kong B (2013) PAX8 is a novel marker for differentiating between various types of tumor, particularly ovarian epithelial carcinomas. Oncol Lett 5:735–738. 10.3892/ol.2013.1121 23425942 10.3892/ol.2013.1121 PMC3576179

[53] Zannini M, Francis-Lang H, Plachov D, Di Lauro R (1992) Pax-8, a paired domain-containing protein, binds to a sequence overlapping the recognition site of a homeodomain and activates transcription from two thyroid-specific promoters. Mol Cell Biol 12:4230–4241. 10.1128/mcb.12.9.4230 1508216 10.1128/mcb.12.9.4230-4241.1992 PMC360331

[54] Zhang P, Zuo H, Nakamura Y, Nakamura M, Wakasa T, Kakudo K (2006) Immunohistochemical analysis of thyroid-specific transcription factors in thyroid tumors. Pathol Int 56:240–245. 10.1111/j.1440-1827.2006.01959.x 16669872 10.1111/j.1440-1827.2006.01959.x

[55] Zhu B, Rohan SM, Lin X (2020) Cytomorphology, immunoprofile, and management of renal oncocytic neoplasms. Cancer Cytopathol 128:962–970. 10.1002/cncy.22330 32697415 10.1002/cncy.22330

