# Supplementary figures and images for: PAX8 expression in cancerous and non-neoplastic tissue: a tissue microarray study on more than 17,000 tumors from 149 different tumor entities
Source: Virchows Arch. 2024 Aug 6;485(3):491–507. doi: 10.1007/s00428-024-03872-y (PMC11415470; doi:10.1007/s00428-024-03872-y)

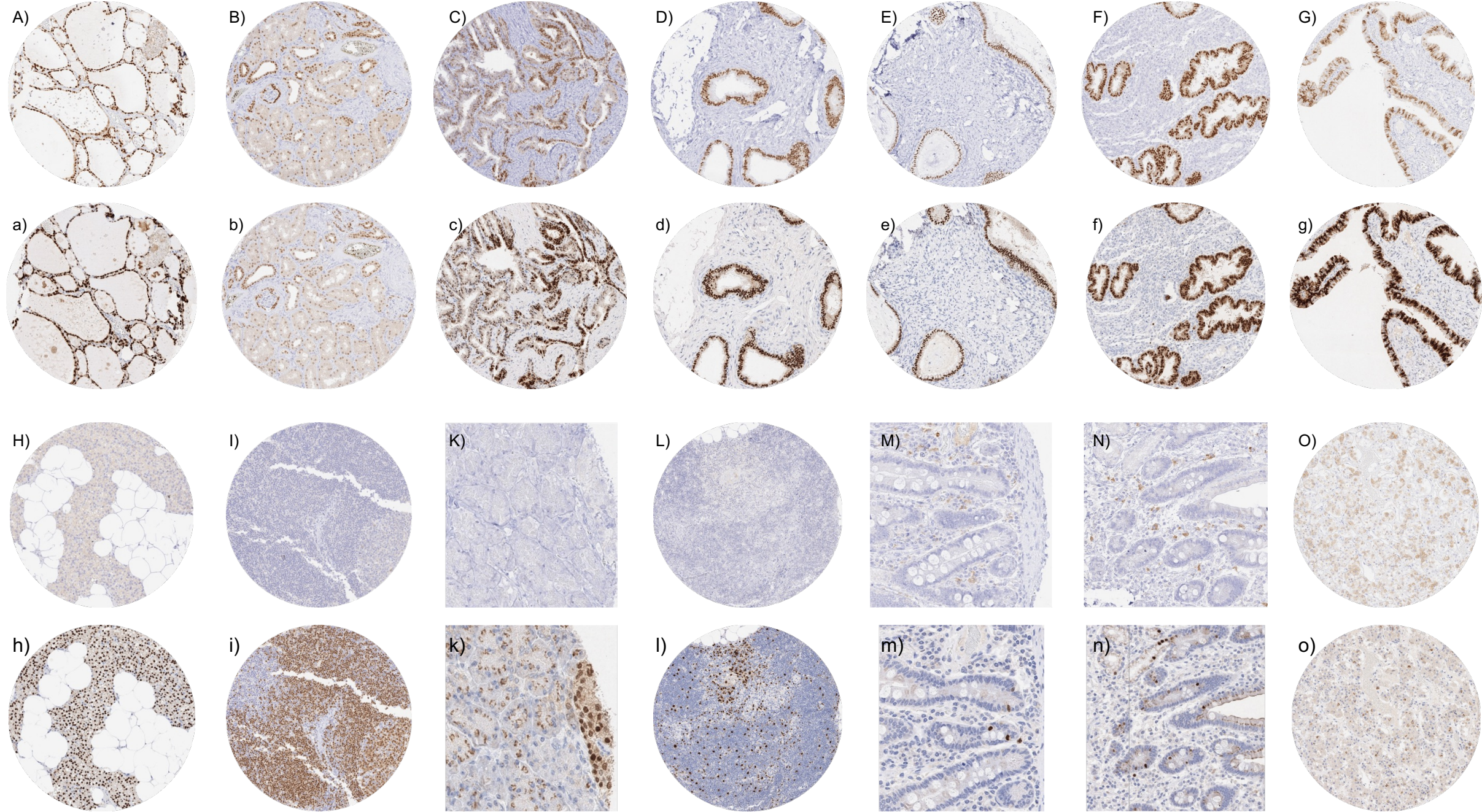

Supplement: Supplementary file 1 — Supplementary file1 (Immunohistochemistry (IHC) validation by comparison of antibodies. The panels show a comparison of IHC results obtained by two independent PAX8 antibodies (MSVA-708R, MRQ-50). Using MSVA-708R, a nuclear PAX8 positivity of variable intensity was seen in distinct cell types of the thyroid (A), kidney (B), seminal vesicle (C), caput epididymis (D), endocervix (E), endometrium (F) and the fallopian tube (G) while staining was absent in parathyroid (H), lymph node (I), pancreas (K), and thymus (L). A purely cytoplasmic staining was seen by MSVA-708R in some inflammatory cells of the gastrointestinal mucosa (M, N) and in some epithelial cells of the adenohypophysis (O). Using clone MRQ-50, a nuclear staining of identical cell types was seen in thyroid (a), kidney (b), seminal vesicle (c), epididymis (d), endocervix (e), endometrium (f) and the fallopian tube (g). In addition, MRQ-50 showed a nuclear staining of parathyroidal epithelial cells (h), a subset of lymphocytes in the lymph node (i), islet cells of the pancreas (and a granular cytoplasmic staining of acinar cells; k), lymphocytes and a subset of epithelial cells of the thymus (l), and of neuroendocrine cells in the gastrointestinal mucosa (m, n) while staining was absent in the adenohypophysis (o). (PDF 1643 KB) [file 428_2024_3872_MOESM1_ESM.pdf]

a)

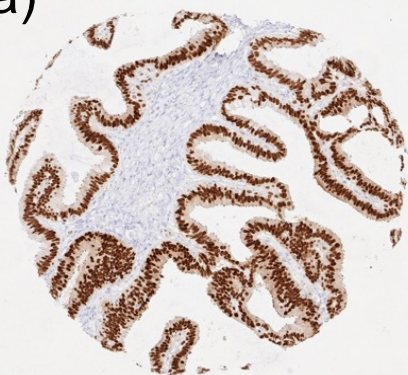

b)

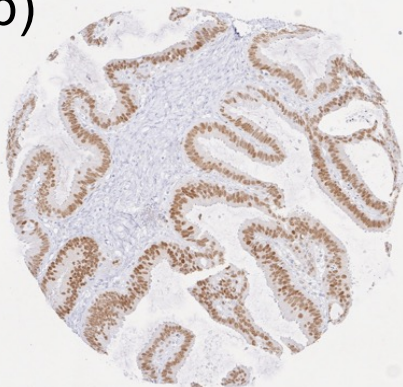

c)

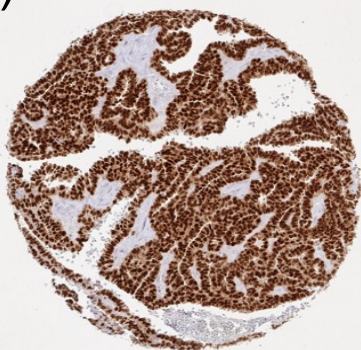

d)

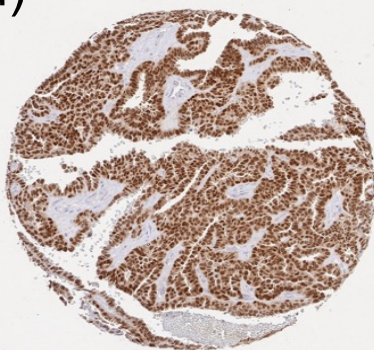

e)

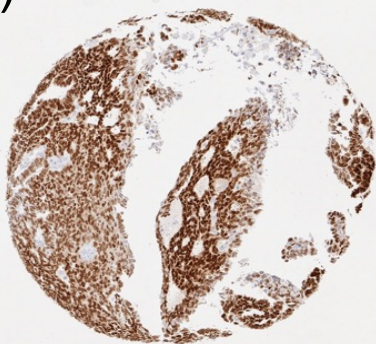

f)

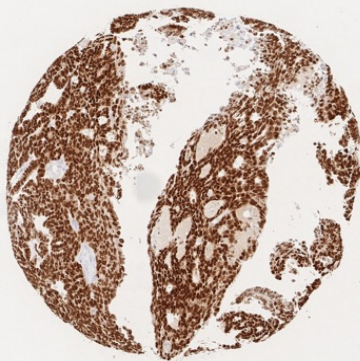

g)

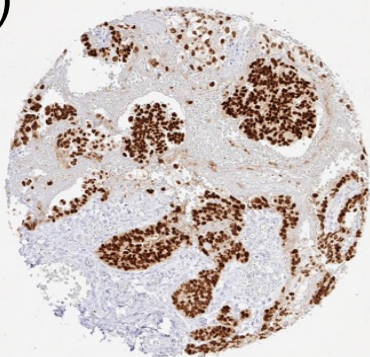

h)

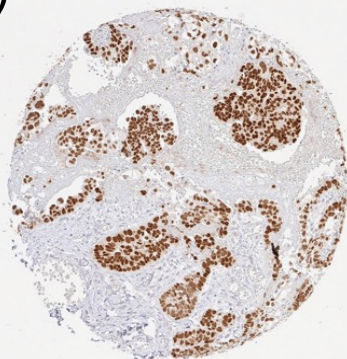

Supplement: Supplementary file 2 — Supplementary file2 (Comparison of anti-PAX8 antibody clones MSVA-708R (left hand side) and MRQ-50 (right hand side) in cancer tissues: Examples of concordant staining. Strong nuclear PAX8 staining is found with both antibodies at comparable frequencies in endometrioid (a,b), serous (c,d), and mucinous ovarian carcinomas (e,f) as well as in carcinosarcomas of the ovaries (g,h). (PDF 506 KB) [file 428_2024_3872_MOESM2_ESM.pdf]

a)

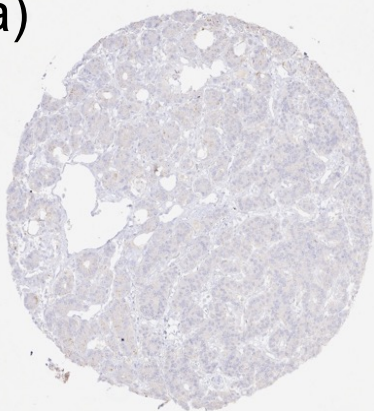

b)

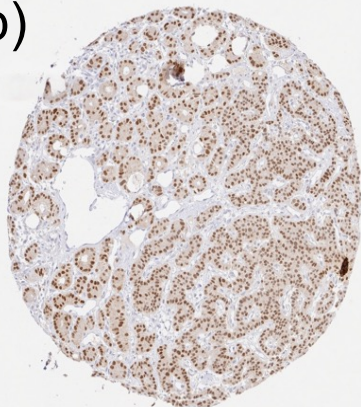

c)

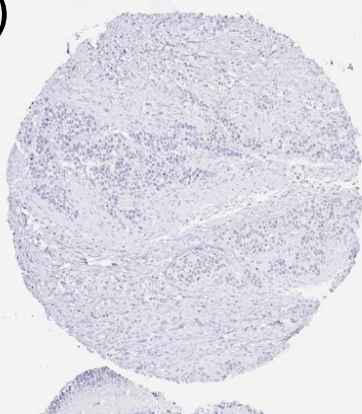

d)

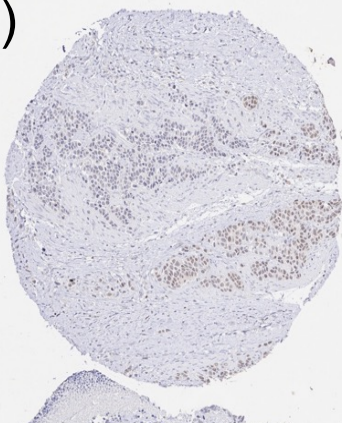

e)

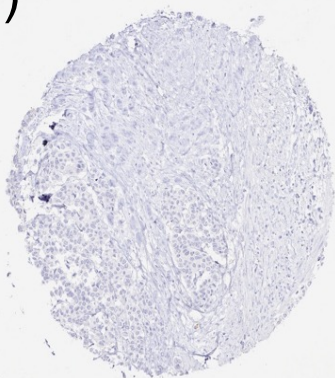

f)

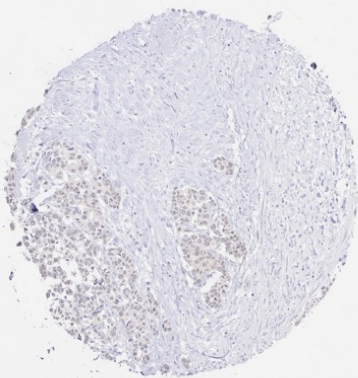

g)

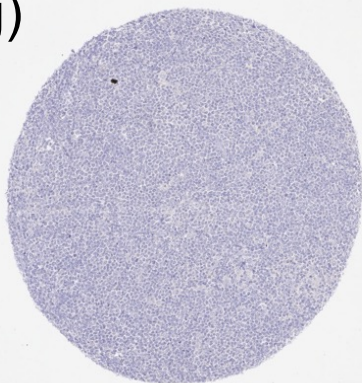

h)

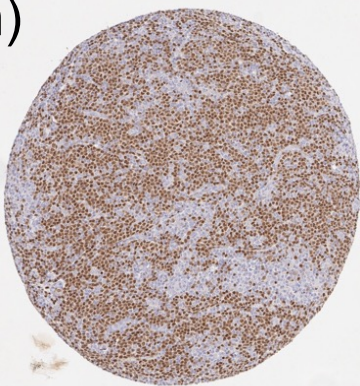

Supplement: Supplementary file 3 — Supplementary file3 (Comparison of anti-PAX8 antibody clones MSVA-708R (left hand side) and MRQ-50 (right hand side) in cancer tissues: Examples of discordant staining. Strong nuclear staining with MRQ-50 in a neuroendocrine tumor of the pancreas (b), in neuroendocrine carcinomas of the colon (d), and gallbladder (f) and in a follicular lymphoma (h). These tumor types stain entirely negative with MSVA-708R (a, c, e, g). (PDF 504 KB) [file 428_2024_3872_MOESM3_ESM.pdf]

0 20 40 60 80 100 %

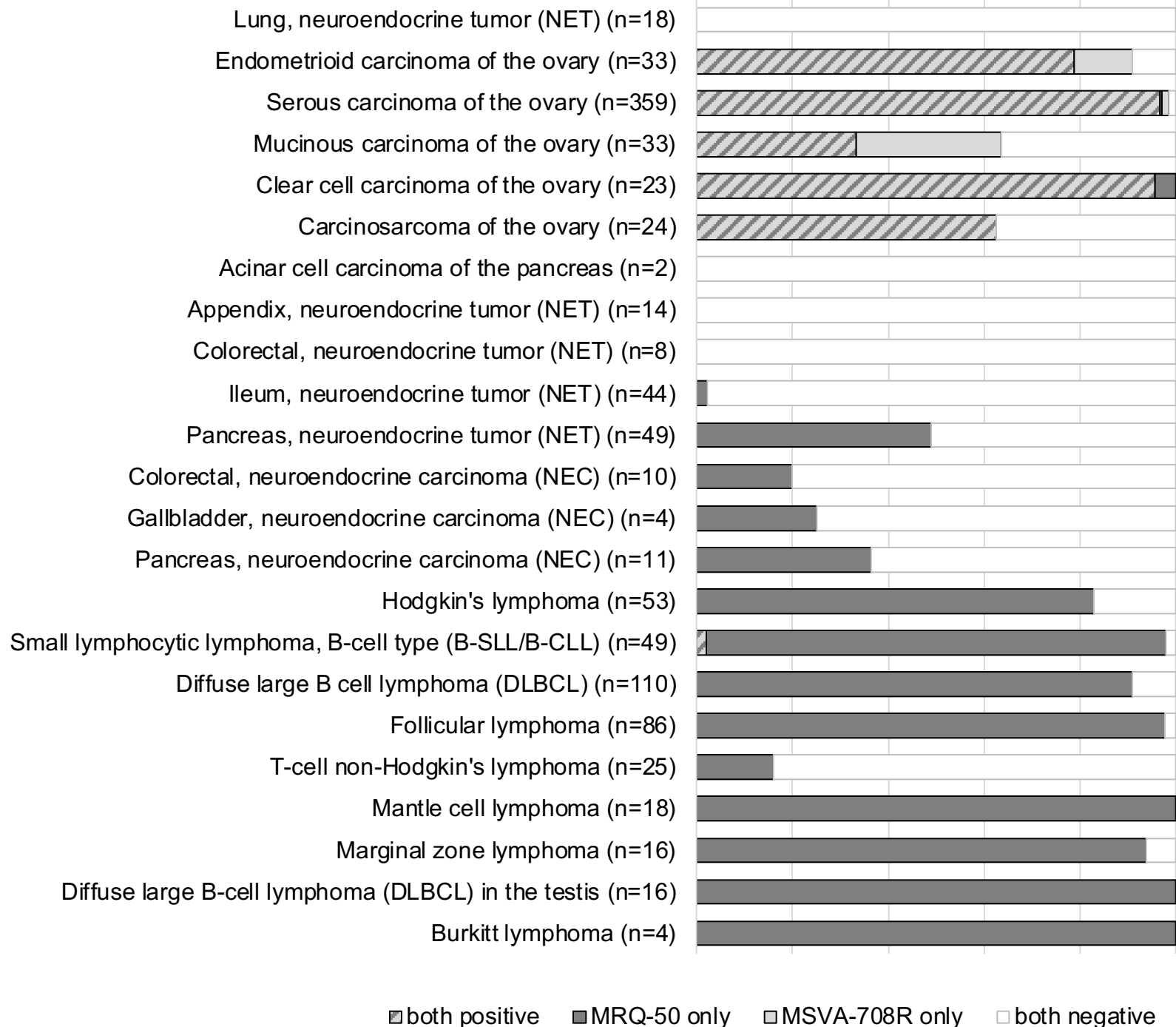

Supplement: Supplementary file 4 — Supplementary file4 (Comparison of PAX8 antibodies in 23 tumor types. (PDF 16 KB) [file 428_2024_3872_MOESM4_ESM.pdf]

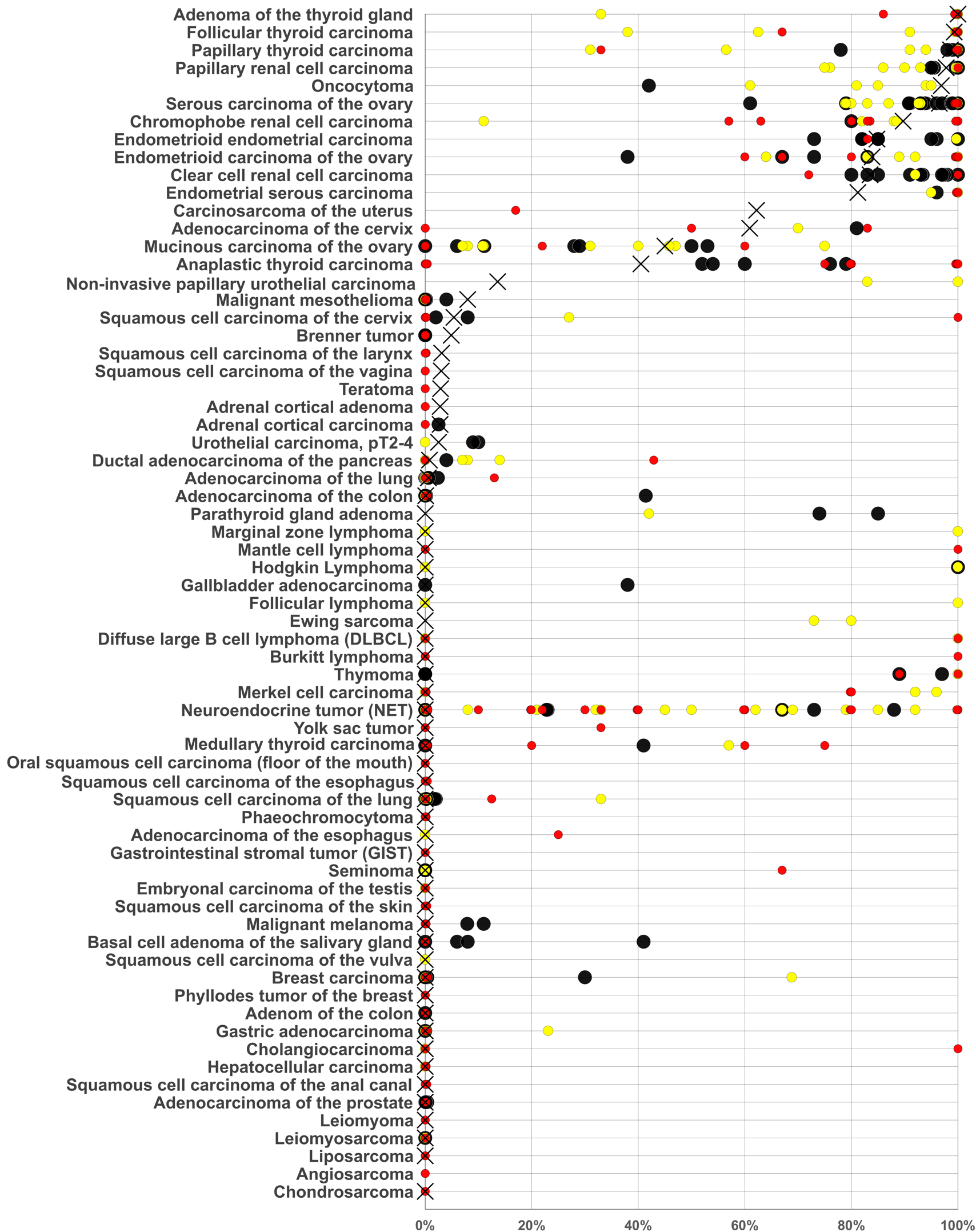

Supplement: Supplementary file 5 — Supplementary file5 (Comparison with previous PAX8 literature. An „X“ indicates the fraction of PAX8 positive cancers in the present study, dots indicate the reported frequencies from the literature for comparison: red dots mark studies with ≤ 10 analyzed tumors, yellow dots mark studies with 11 to 25 analyzed tumors, and green dots mark studies with > 25 analyzed tumors. References are found in supplementary table 2. (PDF 49 KB) [file 428_2024_3872_MOESM5_ESM.pdf]
